# Amelioration of Experimental Acute Pancreatitis with Dachengqi Decoction via Regulation of Necrosis-Apoptosis Switch in the Pancreatic Acinar Cell

**DOI:** 10.1371/journal.pone.0040160

**Published:** 2012-07-02

**Authors:** Jia Wang, Guangyuan Chen, Hanlin Gong, Wei Huang, Dan Long, Wenfu Tang

**Affiliations:** 1 Department of Integrated Traditional Chinese and Western Medicine, West China Hospital, Sichuan University, Chengdu, PR China; 2 Physiological Laboratory, University of Liverpool, Liverpool, United Kingdom; 3 Department of Laboratory of Transplant Engineering and Immunology, West China Hospital, Sichuan University, Chengdu, PR China; University of Valencia, Spain

## Abstract

Severity of acute pancreatitis contributes to the modality of cell death. Pervious studies have demonstrated that the herb medicine formula “Dachengqi Decoction” (DCQD) could ameliorate the severity of acute pancreatitis. However, the biological mechanisms governing its action of most remain unclear. The role of apoptosis/necrosis switch within acute pancreatitis has attracted much interest, because the induction of apoptosis within injured cells might suppress inflammation and ameliorate the disease. In this study, we used cerulein (10^−8^ M)-stimulated AR42J cells as an *in vitro* model of acute pancreatitis and retrograde perfusion into the biliopancreatic duct of 3.5% sodium taurocholate as an *in vivo* rat model. After the treatment of DCQD, cell viability, levels of apoptosis and necrosis, reactive oxygen species positive cells, serum amylase, concentration of nitric oxide and inducible nitric oxide syntheses, pancreatic tissue pathological score and inflammatory cell infiltration were tested. Pretreatment with DCQD increased cell viability, induced apoptosis, decreased necrosis and reduced the severity of pancreatitis tissue. Moreover, treatment with DCQD reduced the generation of reactive oxygen species in AR42J cells but increased the concentration of nitric oxide of pancreatitis tissues. Therefore, the regulation of apoptosis/necrosis switch by DCQD might contribute to ameliorating the pancreatic inflammation and pathological damage. Further, the different effect on reactive oxygen species and nitric oxide may play an important role in DCQD-regulated apoptosis/necrosis switch in acute pancreatitis.

## Introduction

Acute pancreatitis (AP) is an inflammatory condition that its severe form involves systemic inflammatory response syndrome (SIRS) and multiple organ dysfunction syndromes (MODS) [1], [2]. The severity of AP depends largely upon the balance between two forms of cell death-apoptosis and necrosis, the former presumed to be predominantly protective with mild or no inflammatory response, while necrosis, cell membrane integrity is lost, associated with the release of the digestive enzymes and inflammatory mediators, which can ultimately escalate local and systemic damage [3], [4]. A therapeutic agent that could induce apoptosis of injured pancreatic acinar cells by regulating the apoptosis/necrosis switch is likely to reduce necrosis and provide a new effective treatment [3], [5].

Free radicals are molecules produced continuously in cells by several mechanisms and responsible for a wide variety of diseases or conditions. It has been shown that reactive oxygen and nitrogen species (ROS and RNS) contribute to the acinar cell damage during the early phases of pancreatitis [6]. ROS can act as a molecular trigger to activate oxidant-sensitive nuclear transcription factor kappa B (NF-κB) and thus induces cytokine expression, participating in various inflammatory processes. Moreover, an important link between ROS generation and apoptosis has been shown in both human and experimental pancreatitis. Numerous studies have shown many anti-oxidant treatments significantly reduce pancreatic injury and inflammation [7].

In AP, cytokines and other mediators derived from the inflamed pancreas activate the production of the inducible nitric oxide synthase (iNOS). An enhanced formation of nitric oxide (NO) due to the induction of iNOS may be an important factor in the systemic and local haemodynamic disturbances and regulation of pancreatic exocrine secretion associated with AP. Excess of NO cause hypotension and decrease blood perfusion of various organs, including the pancreas and lung, correlating with apoptotic changes [8]. Therefore, treatments that could regulate free radicals ROS or RNS may directly contribute to the modality of acinar cell death and the degree of inflammation.

Dachengqi Decoction (DCQD) composed of *Radix et Rhizoma Rhei* (Dahuang), *Cortex Magnoliae Officinalis* (Houpu), *Fructus Aurantii Immaturus* (Zhishi) and *Natrii Sulphas* (Mangxiao) is traditionally used as the representative prescription purgative for the treatment of constipation and for clearing internal heat in the stomach and intestine [9]. In China, DCQD has been used to treat AP for over 30 years [10]. Recent studies have shown that DCQD can promote gastrointestinal motility, inhibit cytokine activity and immune inflammatory response in AP [11]–[13]. However, most of its biological activities have been studied individually on its ingredients. Studies designed to test the molecular mechanisms of the compound herb formula DCQD in the modality of pancreatic acinar cell death have not been elucidated to date.

Thus, in our present work, we studied the effect of DCQD in regulating the inflammatory response via selective induction of pancreatic acinar cell apoptosis and explored the regulation mechanism of apoptosis/necrosis switch through its opposite effect in regulating ROS and NO *in vitro* and *in vivo*.

## Materials and Methods

### 1. Materials

#### 1.1. Drugs and reagents

The spray-dried *Radix et Rhizoma Rhei*, *Cortex Magnoliae Officinalis*, *Fructus Aurantii Immaturus* and *Natrii Sulphas* powder were purchased from Chengdu Green Herbal Pharmaceutical Co. Ltd (Chengdu, China). The spray-dried powder was mixed of an equal amount and reconstituted with sterile distilled water at concentrations for the crude drug of 2 g/mL DCQD *in vivo* study [14]. *In vitro* study, the mixed powder was reconstituted with PBS to prepare a 50 mg/mL stock solution in dimethylsulfoxide (DMSO) and kept in −20°C. Before being added to cells, the DCQD stock solution was diluted with PBS to prepare the working solutions. The final DMSO concentrations were all less than 0.1% when DCQD was added to cells. The dose of DCQD was calculated and diluted according to its contents quantitatively analyzed by HPLC system. Fetal bovine serum (FBS) was obtained from HyClone (Logan, UT). DMSO, Cerulein, F12K medium and DCFH-DA were obtained from Sigma (St. Louis, MO, USA).

#### 1.2. Rats

Sprague-Dawley rats (243±18 g) were purchased from the Experimental Animal Center of West China Center of Medical Sciences of Sichuan University. All animal studies were performed according to the Guide for the Care and Use of Laboratory Animals of the National Institutes of Health. The protocol was approved by the Committee on the Ethics of Animal Experiments of the Sichuan University.

### 2. Methods

#### 2.1. Cell culture

Rat pancreatic acinar AR42J cells (ATCC, Rockville, MD, USA) were cultured in F12K medium containing 20% FBS and 100 U/mL penicillin, 100 µg/mL streptomycin in standard condition (37°C and 5%CO_2_). All experiments were carried out 24 h after cells were seeded. To investigate the protective effects of DCQD against AP, AR42J cells were treated with or without DCQD prior for 30 min, then further co-incubated with cerulein (10^−8^ M) for another 24 h.

#### 2.2. Cell viability assay

Cell survival was assessed by WST viability assay kit containing WST-8(2-(2-methoxy-4-nitrophenyl)-3-(4-nitrophenyl)-5-(2,4-disulfophenyl)-2H-tetrazolium, monosodium salt) according to the manufacturer's protocol (Roche, Basel, Switzerland). AR42J cells were plated in 96-well plates at 2×10^4^ cells/well. After 24 h incubation, cells were pretreated with or without DCQD at different concentrations (0–0.004 g/mL) and then were further co-incubated with cerulein for 24 h, WST-8 solution (0.5 mg/mL) was added to each well and incubated at 5% CO_2_ 37°C for 2 h. The cell viability was determined by the differences absorbance at wavelengths of 450 nm and 630 nm. The relative cell viability rate was calculated according to the following formula: Cell viability rate (%) = 100%×mean absorbance of cells in sample groups/mean absorbance for cells in control group.

#### 2.3. LDH assay

Necrotic cell death was assessed by the release of lactate dehydrogenase (LDH) from the cytosol of the damaged cells into the supernatant [15], using the LDH cytotoxicity detection kit (Nanjing Jiancheng Bioengineering Institute, Nanjing, China) for various time point 0–24 h according to the manufacturer's instructions. Values for LDH release are presented as the percentage of total cellular LDH from the following equation [16]: LDH release (%) = total extracellular LDH activity at the time point×100/total LDH activity.

#### 2.4. Apoptosis assay

Cells were stained with Annexin V-FITC Apoptosis detection kit (Nanjing Kaiji, Nanjing, China) following the manufacturer's instructions to detect early apoptotic cells (Annexin V+PI− events) and necrotic or late apoptotic cells (Annexin V+PI+) by flow cytometry. Briefly, AR42J cells were treated or not with DCQD prior for 30 min and then stimulated with cerulein (10^−8^ M) for 24 h. Then cells were collected and resuspended in the culture medium at a density of 1×10^6^ cells/mL, stained with 5 µL of Annexin V-FITC and 5 µL propidium iodide (PI) in 300 µL binding buffer (10 mM HEPES, pH 7.4, 140 mM NaOH, and 2.5 mM CaCl_2_) according to the manufacturer's instructions for 15 min at room temperature in the dark. Quantification of apoptotic cells was analysis by flow cytometry (FACScan, Becton Dickinson, USA).

#### 2.5. Measurement of ROS generation

The generation of ROS in cells was determined using a FACScan flow cytometry following the manufacturer's instructions. Briefly, AR42J cells were pretreated with DCQD 30 min before stimulated with cerulein for 24 h. Cells were collected and incubated with 10 µM/L DCFH-DA 30 min in the dark and then washed twice with PBS. Intracellular low-molecular-weight peroxides oxidize DCFH-DA to the highly fluorescent compound dichlorofluorescein (DCF). Then the cells were harvested and the pellets were suspended in 300 µl PBS at an excitation wavelength of 488 nm and an emission wavelength of 525 nm.

#### 2.6. Animal models and treatment with DCQD

Sprague-Dawley rats were divided randomly into sham-operated group, AP group and DCQD- treated group (n = 6). While the rats were under ether anesthesia and laparotomy, pancreatitis was induced by retrograde perfusion into the biliopancreatic duct of 3.5% sodium taurocholate (Sigma, St. Louis, MO, USA) (1 mL/kg body weight) at a rate of 0.2 mL/min with a microinfusion pump [17]. The entire procedure from induction of anesthesia to closure of the incisions requires ∼30 min for each animal. The same procedure was applied to sham-operated group but receiving an intraductal perfusion of saline (NaCl 0.9%) instead of sodium taurocholate. In DCQD- treated group, the rats recovered from anesthesia and were administered intragastrically DCQD 20 g/kg body weight (equivalent to 2 g/mL crude herbs) 2 h after operation. In the sham-operated group and AP group, rats were given equal volume of saline. After 48 h, blood were obtained from the vena caudalis and centrifuged to obtain serum for amylase examination. The animals were sacrificed by exsanguination while under ether anesthesia and the pancreatic tissues were rapidly collected for pathological and apoptotic examinations. Tissue homogenate was collected for NO and iNOS concentration measurement.

#### 2.7. Amylase and NO and iNOS Assay

Serum was collected from the rats for amylase activity (U/L) measurement by an enzymatic assay kit from Sigma (St. Louis, MO, USA) according to manufacturer's instructions. Tissue homogenate was collected for NO and iNOS concentration measurement, using nitric oxide and inducible nitric oxide synthetase assay kits (Nanjin Jiancheng Biological engineering Company, Nanjin, China) according to manufacturer's instructions.

#### 2.8. TUNEL assay

The apoptotic cells in tissue samples were detected using an In Situ Cell Death Detection kit (Roche, Switzerland) according to the manufacturer's instructions. Briefly, paraffin embedded specimens were cut into 4–5 µm thickness sections. After deparaffinization and washed in PBS, The sections were treated with proteinase K, then incubated with TUNEL reaction mixture at 37°C for 1 h. Slides were washed with PBS and then treated with HRP and DAB terminated until the color was developed. Apoptotic index was determined by counting the number of TUNEL-positive cells. Eight slides per block were evaluated. For each slide, 8 fields were randomly chosen, and `100 cells per field were counted and calculated the apoptosis index.

#### 2.9. Histopathologic analysis of pancreas

At the end of experiment, pancreatic tissues were promptly collected, fixed in 10% neutral formalin and embedded in paraffin. The paraffin-embedded tissue blocks were cut into 5 µm thick sections and stained with hematoxylin and eosin. Specimens were graded by two independent pathologists blinded to the experimental setup using a scoring system for the extent and severity of pancreatitis (0–4, normal to severe, respectively), the degree of interstitial edema, hemorrhage, hyperemia, necrosis, leukocyte infiltration of the pancreatic tissue as previously described [18], [19].

#### 2.10. Leukocyte infiltration assay

For evaluation of the infiltration number of leukocyte during pancreatitis we randomly chose 8 to 10 consecutive high-power fields for each rat (n = 6) on a scale of 0–4 by two researchers in a blinded manner as previously described [18], [19].

### 3. Statistical analysis

Statistical analysis was carried out using the PEMS3.1 statistical program. All data represent at least three independent experiments and are expressed as the mean ± standard errors of mean (S.E.M.). One-way repeated-measures ANOVA (followed by multiple pair-wise comparisons using Student-Neuman-Keuls procedure) were used for the analysis of differences between the experimental and control groups. Values of *P*<0.05 were regarded as statistically significant.

## Results

### 1. DCQD enhanced cerulein-induced AR42J cell viability

**Figure 1:**
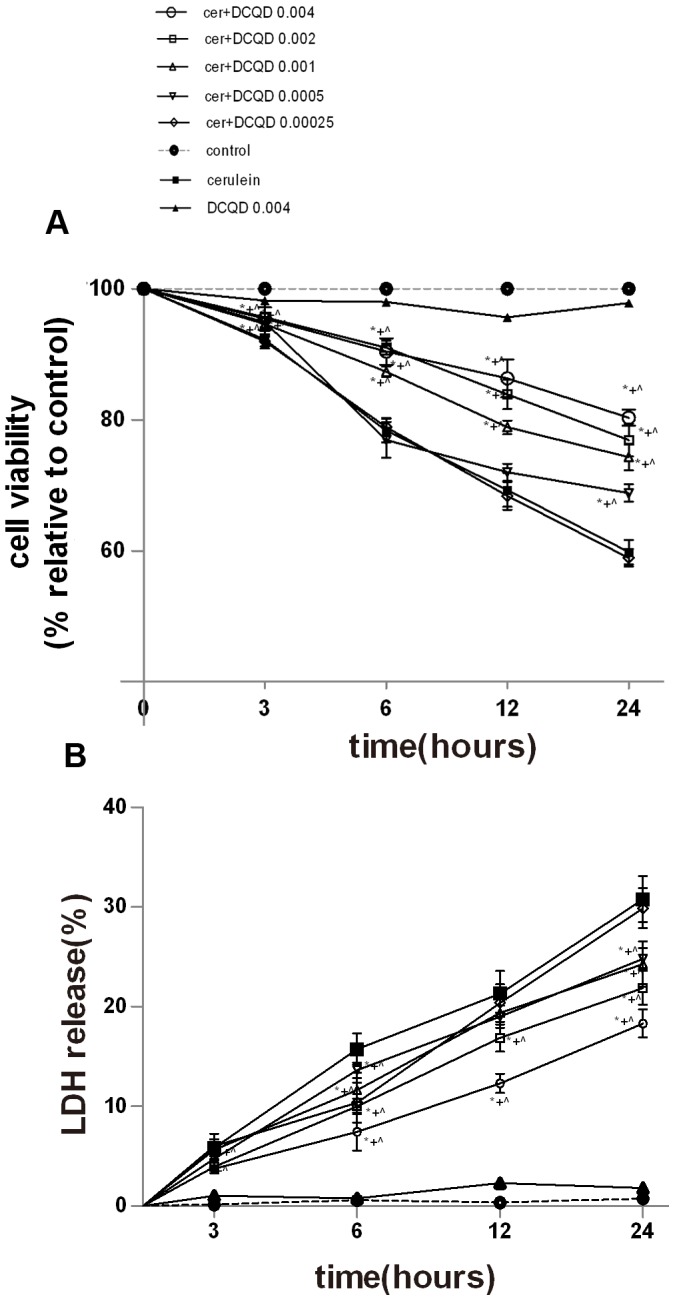
Effects of DCQD on the reduction of cerulein-induced necrosis of AR42J cells. The cells were pre-treated with increasing concentrations of DCQD (0.00025 g/mL, 0.0005 g/mL, 0.001 g/mL, 0.002 g/mL and 0.004 g/mL) for 30 min and then co-incubated with or without 10^−8^ M cerulein for another 0–24 h. (A) Cell viability rate was examined using WST-8 assay. (B) Necrotic cell death rate was assessed by the release rate of LDH. The results are mean ± SE (n = 5) for three independent experiments. *P<0.05 versus control group; +*P*<0.05 versus DCQD 0.004 g/mL group; ^*P*<0.05 versus cerulein group.

In order to examine the effect of DCQD on cell viability, pancreatic acinar AR42J cells were treated with increasing concentrations of DCQD (0.00025 g/mL, 0.0005 g/mL, 0.001 g/mL, 0.002 g/mL and 0.004 g/mL) for 0–24 hours based on the components' concentrations of DCQD in blood of our previous study [20], [21]. As shown in Figure 1A, there were few dead cells present in the control group, and the cell viability significantly decreased after cerulein was added. Treatment of AR42J cells with DCQD for 24 hours caused a concentration-dependent protective effect, and the maximum effect was obtained at the dose of 0.004 g/mL. The viability of cells pretreated with 0.004 g/mL DCQD for 24 h increased significantly compared to the cerulein stimulated group. One characteristic of pancreatic acinar cell stimulated with supramaximal doses of cerulein is the induction of necrosis [16]. The process of necrosis damages the plasma membranes, and release LDH into the extracellular medium. To evaluate necrosis in the present study, we measured the release of LDH from the damaged AR42J cells following 24 h treatment with cerulein. The release of LDH in the control group was at relatively lower levels, and the levels of LDH significantly increased after the addition of cerulein and different concentrations of DCQD. The level of necrotic cells was decreased after the pretreatment of DCQD with increased concentration. In our study, supramaximal cerulein treatment significantly increased LDH release from pancreatic acinar cells. However, pretreatment with 0.004 g/mL DCQD significantly diminished LDH release compared to the cerulein-stimulated cell AP model group at 24 h (Figure 1B).

### 2. DCQD induced pancreatitis AR42J cells apoptosis

**Figure 2:**
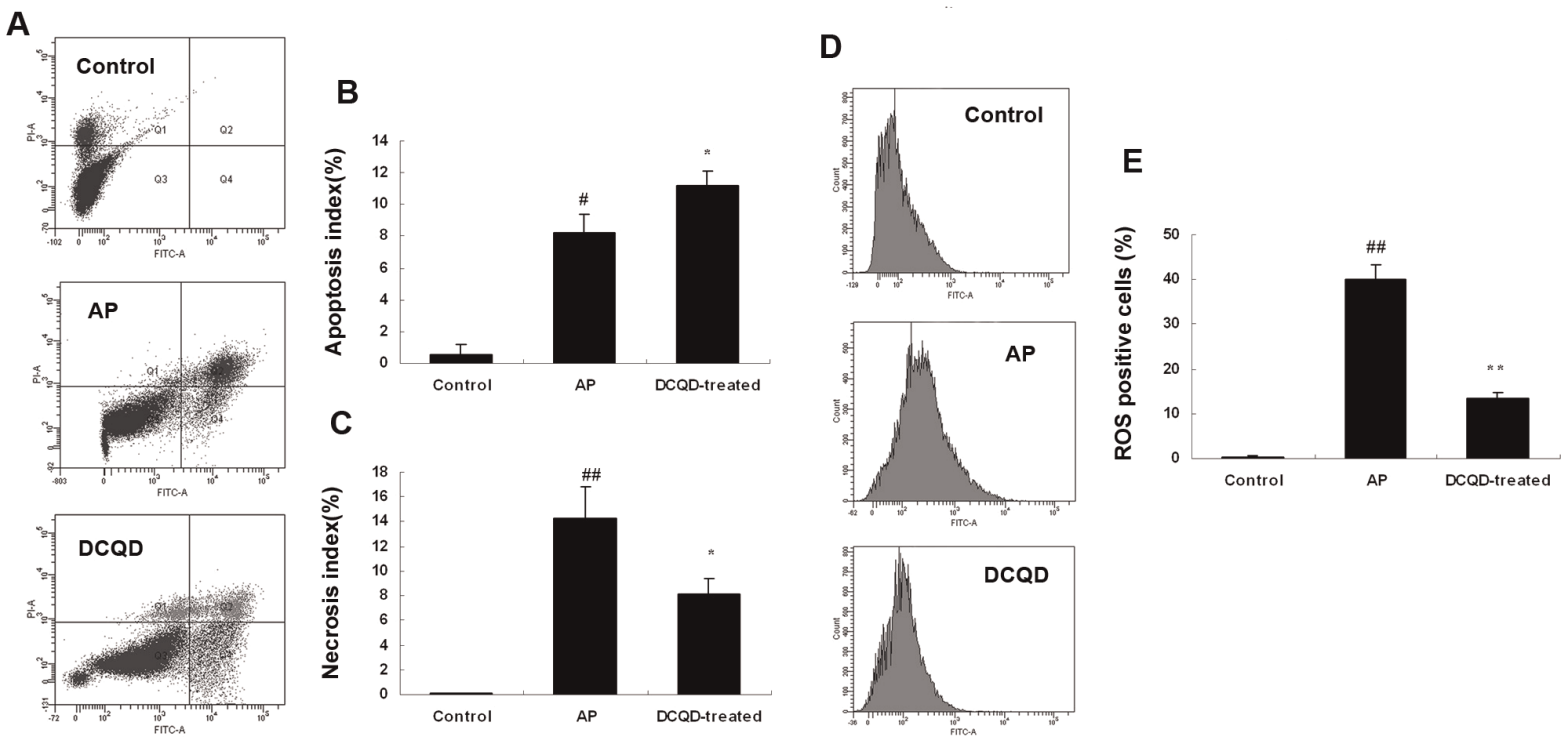
DCQD regulated cerulein-induced AR42J necrosis-apoptosis switch through ROS. The cells were pre-treated with 0.004 g/mL DCQD for 30 min and then co-incubated with or without 10−8 M cerulein for another 24 h. (A) Flow cytometry analysis of the apoptotic and necrotic cells among the AR42J cells. Four different regions can be found in each panel in the figure for flow cytometry detection: Viable cells (Annexin V−/PI−) are located in the lower left, early apoptotic cells (Annexin V+/PI−) in the lower right, late apoptotic and necrotic cells (Annexin V+/PI+) in the upper right and primary necrotic cells (Annexin V−/PI+) in the upper left quadrants, respectively. (B) The percentages of apoptotic cells and (C) necrotic cells were compared. (D) Generation of ROS in AR42J cells were detected by DCF using flow cytometry (E) and ROS positive cells were compared. The results are mean ± SE for three independent experiments. ^#^ *P*<0.05 and ^##^ *P*<0.01 versus control cells; ^*^ *P*<0.05 and ^**^ *P*<0.01 versus AP group.

To determine the effects of inducing apoptosis by DCQD on AR42J cells, we further analyze apoptosis using Annexin V/PI staining. The Annexin V−/PI− population was regarded as normal healthy cells, while Annexin V+/PI− cells were taken as a measure of early apoptosis and Annexin V+/PI+ as necrosis/late apoptosis. Our results showed that there was a very low level of cell death in the control group, 24 h treatment with cerulein significantly increased cell death (Figure 2A). In the AP group, there were fewer apoptotic cells but more necrotic cells (Figure 2B). After pretreated with DCQD, the number of apoptotic cells increased and the number of necrotic cells decreased significantly comparing with AP group cells at 24 h (Figure 2C).

### 3. DCQD reduced ROS in cerulein-induced AR42J cells

Acinar cellular damage induced by supramaximal cerulein could originate from premature intracellular enzyme activation, but also from injurious levels of ROS. We explored whether DCQD could diminish the supramaximal cerulein-induced necrosis by interfering with ROS production. The ROS positive cells pretreated with or without DCQD before stimulated with cerulein for 24 h were analyzed (Figure 2D). A very low level of ROS positive cells were detected in the control group, but in AP group ROS positive cells significantly increased. DCQD pretreated pancreatic acinar cells before cerulein stimulating significantly decreased ROS positive cells remarkably (Figure 2E).

### 4. DCQD reduced the release of serum amylase in the rats' model of AP

**Figure 3:**
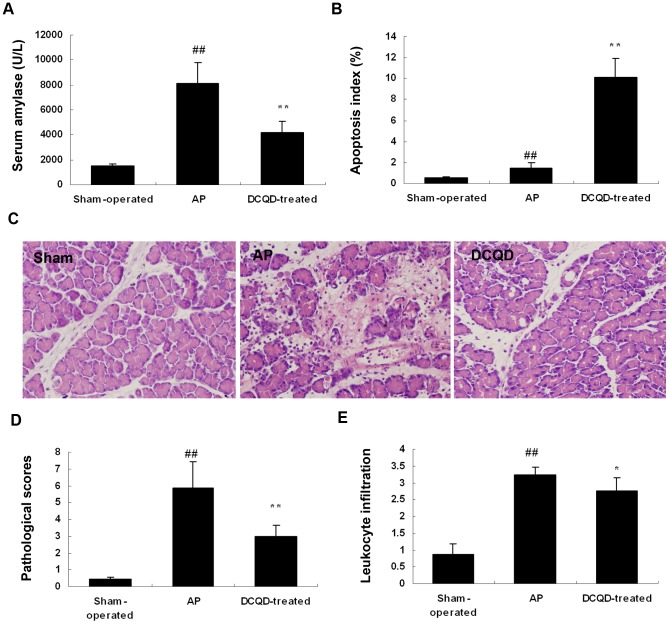
DCQD alleviated acute pancreatitis-associated tissue damage. Rats (n = 6 per group) were given DCQD (20 g/kg body weight) 2 h after operation. After 48 h, the blood was obtained for amylase examination. The pancreatic tissues were collected for examination of pathological and apoptotic examinations (A) Serum amylase activity assay. (B) TUNEL-positive cells percentage in the pancreatic tissue. (C) Pathological changes of pancreas in different groups observed by HE staining (Light microscopy, ×400). Sham-operated group rats showed slightly edematous. Compared with sham-operated rats, AP group rats showed a severe degree of pancreatic damage with edema, hemorrhage, necrosis, pancreatic acinar cell vacuolization and infiltration of inflammatory cells. In DCQD-treated group, rats showed a reduction of edema, hemorrhage, necrosis, and inflammatory cells infiltration. (D) Pathological scores of pancreatic injury (E) and leukocyte infiltration counted in 8 to 10 consecutive high-power fields per slide of Sham-operated group, AP group and DCQD-treated group. The results are mean ± SE for three independent experiments. ^#^ *P*<0.05 and ^##^ *P*<0.01 versus sham-operated group; ^*^ *P*<0.05 and ^**^ *P*<0.01 versus AP group.

Sodium taurocholate stimulation caused a statistically significant increase of serum amylase at 48 h in the AP group compared with the sham-operated group *in vivo*. In DCQD-treated group, the serum amylase values were significantly lower than that in the AP group (Figure 3A).

### 5. DCQD induced apoptosis of pancreatitis acinar cells determined by TUNEL staining

TUNEL-stained slides revealed that pancreas tissue from sham-operated rats exhibited very low levels of apoptosis. In contrast, a significant number of TUNEL-positive cells were detected in pancreas tissue within the AP group and DCQD- treated group at 48 h. Treatment with DCQD significantly increase the percentage of TUNEL-positive cells compared with the AP group (Figure 3B). This result indicates that DCQD may preferentially induce apoptosis within injured cells, which is consistent with our *in vitro* study.

### 6. DCQD alleviated the severity of experimental AP

In the sham-operated group, the pancreas was few edematous, with the infiltration of a few inflammatory cells but without obvious hemorrhage, necrosis of acinar cells or the adjacent fat tissues. However, the AP group showed the features of a severe form of AP characterized by expansion of interlobular and interlobular spaces caused by moderate to severe interstitial edema, extensive infiltration with inflammatory cells, obvious pancreatic acinar cells vacuolization, necrosis and hemorrhage (Figure 3C). The rats treated with DCQD showed a significant reduction of inflammatory cells infiltration, hemorrhage, necrosis and interstitial edema compared to the AP group. The standard pathological scores in both AP and DCQD-treated groups significantly exceeded the sham-operation group at 48 h. The scores of DCQD-treated group were significantly lower than those of the AP group at 48 hours (Figure 3D).

### 7. DCQD reduced the leukocyte infiltration of pancreatitis tissues

In AP group, there was a significant increase of leukocyte infiltration of pancreas tissue at 48 h after sodium taurocholate stimulation compared with the sham-operated group. In DCQD-treated group, leukocyte infiltration was significantly lower than that in the AP group (Figure 3E).

### 8. DCQD enhanced the acute pancreatitis-associated tissue concentration of NO and iNOS

**Figure 4:**
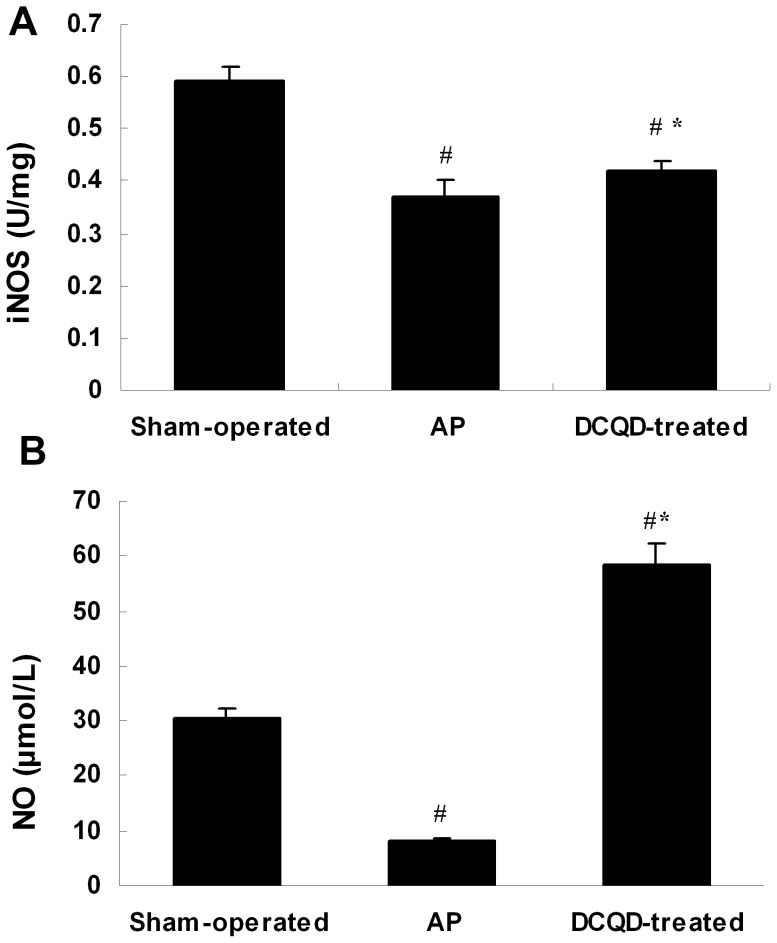
DCQD enhanced the acute pancreatitis-associated tissue concentration of NO and iNOS. Tissue homogenates were collected for NO and iNOS concentration measurement after 48 h treatment. (A) NO concentration (B) and iNOS concentration of sham-operated group, AP group and DCQD-treated group. The results are mean ± SE (n = 6 per group) for three independent experiments. ^#^ *P*<0.01 versus sham-operated group; ^*^ *P*<0.01 versus sham-operated group and AP group.

There were significant differences in the concentration of NO and iNOS among the groups. The results showed that the concentration of NO and iNOS in the AP group were significantly lower than that in the sham-operated group, whereas the concentration of NO and iNOS in the DCQD-treated group were significantly higher than that in the sham-operated group at 48 h (Figure 4). These results suggest that treatment with DCQD could increase the production of NO in pancreatic tissues, which is a major factor correlated with the appearance of apoptosis.

## Discussion

AP is a multifactorial disease associated with the excessive inflammatory response, which can ultimately lead to devastating consequences. The form of cell death determines the severity of AP. Recent studies prove the apoptosis could be a beneficial reaction to AP, because apoptotic cells maintain membrane integrity, therefore, unlike necrotic cells, the leakage of intracellular components containing proinflammatory and immunogenic materials could be prevented actively to avoid inflammatory response [3], [22]. Crucially the two kinds of cell death, necrosis and apoptosis, may be interchangeable under certain conditions [23]. Therefore, if the therapeutic agent could regulate the apoptosis/necrosis switch, it might inhibit the inflammatory response, protecting against progression toward AP.

In some *in vivo* studies, the modified formula of DCQD and its components exhibited potencies in inducing apoptosis of pancreatitis acinar cell [24], [25]. This study shows that DCQD promotes acinar cell apoptosis both *in vitro* and *in vivo* models of AP with a reduction in necrosis, suggesting a switch from deleterious necrotic cell death to a milder apoptotic form. Additionally, pancreatic tissue necrosis, hemorrhage and inflammatory cell infiltration were significantly alleviated in DCQD-treated group *in vivo*. The concentration of LDH in the supernatant of the culture solution can indirectly reflect cell membrane damage resulting in necrosis [26]. It was seen that both the number of necrotic cell and the level of LDH were lower in DCQD-treated group than in the AP group *in vitro*. The plasma concentration of amylase is another parameter for the severity of AP. In this study, its levels were significantly lower in DCQD-treated group than in the AP group *in vivo*. The treatment mechanism of DCQD may occur via inducing apoptosis of injured acinar cells, decreasing necrosis, which help to avoid the release of digestive enzyme and various inflammatory mediators, significantly attenuating the progression of pancreatic injury.

Oxidative stress is regarded as an important determinant of the severity of acute pancreatitis [27]. Large amounts of ROS can directly activate the oxidant-sensitive transcription factor of NF-κB and generate an inflammatory response, which attracts more oxidative stress-generating neutrophils to worsen local tissue destruction and to cause distant organ injury [28], [29]. Moreover, it has been demonstrated that the level of ROS involved in apoptosis/necrosis switch [30], [31]. In our study, there was a lower level of ROS and necrosis but a higher level apoptosis in DCQD-treated group than in AP group *in vitro*, indicating that DCQD have the antioxidant effect to reduce the production of ROS and might switch acinar cells death from necrosis to apoptosis, alleviating subsequent inflammatory response.

The role of NO in the pathogenesis of AP remains controversial. Some studies showed that the proinflammatory cytokines activate the production of the iNOS, resulting in overproduction of NO, which could promote pancreatic injury [32], [33]. Whereas others reported that NO acts as a biological scavenger and inactivates ROS, which protects pancreatic acinar cells [34] and has also beneficial effects by inhibition of neutrophil accumulation and improvement of microcirculation [35], [36]. Our data showed that after treated with DCQD, the production of NO in pancreatic tissues was increased, accompanying the increase of apoptosis with the decrease of inflammatory cells infiltration and pathological scores in pancreatic tissues. This indicated that the increase of NO and iNOS was not the reason of pancreatic tissue damage, but a protective factor might be involved in inducing apoptosis and reduce pancreatic tissue pathological severity.

ROS and nitrogen oxide species play important and different roles in various physiological and pathological states. In our study, DCQD exerting an opposite regulation on NO and ROS may come from its scavenging effects which remain to be understood. These findings provided evidence that treated with DCQD reduce the generation of ROS and increase the NO pancreatitis acinar cells, therefore regulate apoptosis/necrosis switch, induce apoptosis of injured acinar cells and inhibit the subsequent amplifying inflammatory response, which in turn protects against AP.

In the present study, we introduce the ideas and methods of translating research into traditional Chinese medicine, and this is the first time to research the way of compound Chinese herb formula DCQD regulating apoptosis/necrosis switch on AP *in vitro* and *in vivo*. Here we conclude that DCQD could inhibit the local and systematic inflammatory response and alleviate the pancreatic damage via regulating the pancreatic cell necrosis/apoptosis switch. Future study should be directed at signaling pathways of ROS and NO in regulating injured pancreatitis acinar cell apoptosis by the treatment of DCQD.
